# Comparison of a Vibrating Belt Versus a Positional Pillow for the Treatment of Positional Obstructive Sleep Apnea in a Real-World Setting: Results of a Prospective Randomized Crossover Trial

**DOI:** 10.7759/cureus.81666

**Published:** 2025-04-03

**Authors:** Diana Aguilar Pelaez, Sarah Carlier, Thomas Mettay, Ramona Silvia Vulcan, Johan Newell, Anne Violette Bruyneel, Marie Bruyneel

**Affiliations:** 1 Pneumology, Centre Hospitalier Universitaire (CHU) Saint-Pierre, Brussels, BEL; 2 Pneumology, Centre Hospitalier Universitaire (CHU) Brugmann, Brussels, BEL; 3 Sleep Laboratory, Centre Hospitalier Universitaire (CHU) Brugmann, Brussels, BEL; 4 Physiotherapy, Geneva School of Health Sciences, Haute École Spécialisée De Suisse Occidentale (HES-SO), Geneva, CHE

**Keywords:** obstructive sleep apnea, polysomnography, positional obstructive sleep apnea, positional pillow, sleep quality, vibrating belt

## Abstract

Purpose: Obstructive sleep apnea (OSA) is a treatable global health concern with increasing prevalence, driven by the obesity epidemic and the aging population. Unfortunately, approximately one-third of patients are non-adherent to long-term treatment. This highlights the importance of identifying those with positional obstructive sleep apnea (POSA), which affects 25%-35% of the OSA population, as these patients may benefit from alternative therapeutic interventions such as vibrating positional devices or positional pillows. The aim of this prospective randomized crossover study was to assess the efficacy of two different positional therapies (PTs): a positional pillow (Posiform®) and a vibrating belt (Somnofit-belt®), for reducing supine sleep time in POSA patients in the home setting.

Methods: Adults with POSA (n=102), diagnosed by polysomnography (PSG) at two university hospitals, were screened, and 19 patients met the criteria for at-home supine sleep and were included. Patients were randomized to use either the vibrating belt first and then the pillow, or the reverse. Patient questionnaires were used to assess sleep quality and comfort, and the Somnibel Pro® device was used to assess patients’ sleep position.

Results: A statistically significant reduction in supine sleep time was observed in patients using the vibrating belt compared to baseline (p = 0.0001). No difference was observed for the pillow. There was no significant difference in sleep quality or comfort between the devices. This study also highlights the observation that supine sleep is infrequent in the home setting.

Conclusions: In this study, we have shown a significant reduction in supine sleep time in POSA patients using the vibrating belt, which was not observed with the positional pillow. However, the results must be qualified by the small number of patients included.

## Introduction

Obstructive sleep apnea (OSA) syndrome is a growing health problem, related mainly to the obesity epidemic and the aging of the population. In addition, OSA has recently been recognized as an independent risk factor for hypertension, arrhythmia, coronary heart disease, and stroke [1, 2]. The prevalence of OSA is increasing, and it is estimated to affect 13% of men and 6% of women [3]. In a recent epidemiologic study conducted in Switzerland, Heinzer et al. reported that up to 50% of males aged 49-68 years are affected by the disorder [4]. OSA symptoms include daytime sleepiness and nocturnal choking sensations, but the condition can also be associated with numerous other nighttime and daytime symptoms that can impair quality of life [5].

Continuous positive airway pressure (CPAP) remains the cornerstone of treatment for moderate-to-severe OSA and has been shown to provide a survival benefit in patients with severe disease, improve sleep quality and health-related quality of life, and reduce cardiovascular events such as stroke and myocardial infarction [2, 6, 7]. However, achieving adequate adherence remains challenging and depends on several factors, including psychological barriers, social concerns, side effects, healthcare settings, and disease characteristics [8, 9]. A systematic literature review by Rotenberg et al. found that overall CPAP non-adherence was 34.1% across studies conducted over a 20-year period [10].

It is currently recognized that the definition of OSA does not correspond to a single clinical pattern. Based on the presence of excessive daytime sleepiness (EDS), insomnia, or comorbidities, different clinical phenotypes of OSA have been described [11, 12]. Positional obstructive sleep apnea (POSA) is a common clinical phenotype of OSA, referring to patients who exhibit supine-isolated OSA. The prevalence of POSA is approximately 25%-35% of the OSA population, depending on the definition used [13]. Patients with POSA are generally younger and less obese than those with non-positional OSA and typically have mild-to-moderate OSA [14]. The first-line treatment for POSA is not CPAP, but positional therapy (PT), which has been used for decades. The initial approach was simple, involving the placement of a tennis ball in the back of the nightshirt. While this strategy is still used, results are disappointing, with only 6% of patients able to maintain this therapy long-term [15]. Recently, new-generation electronic devices have been introduced. These devices deliver vibrations to the neck or chest to encourage subjects to move away from the supine position [16]. For example, the sleep position trainer (SPT), worn as a simple chest belt, has been shown to be more effective than the tennis ball method and results in better mid-term compliance [17]. A recent meta-analysis reported a 50% reduction in the apnea-hypopnea index (AHI) and an 80% reduction in supine sleep time with the use of vibrating devices [18]. Other treatments have also been tested, such as positional pillows, which require patients to sleep in a lateral position. In a small study, a positional pillow was shown to be effective in reducing AHI and supine sleep time [19]. One of the latest commercially available devices is the Somnibel Pro®, a vibrating postural device placed on the forehead, which also records sleep position during the night. A pilot study of this device reported significant reductions in AHI and supine sleep time [20].

Given the significant number of positional treatments currently available, the aim of this study was to assess the efficacy of two different positional therapies, a positional pillow and a vibrating belt, in reducing supine sleep time for POSA in a home setting.

## Materials and methods

Study design

This was a prospective randomized cross-over trial comparing two different PTs, a positional pillow and a vibrating belt, for the treatment of POSA in the home setting. The study was performed in the sleep lab at two tertiary university hospitals, CHU Brugmann and CHU Saint-Pierre, Brussels, Belgium. All included individuals provided written informed consent to participate in the study. The study protocol was approved by the ethics committee of both hospitals (reference B076201941938). The trial was registered prior to the start of the study: ClinicalTrials.gov identifier: NCT04425408, date of registration: 09/06/2020.

Patients

Adult patients (>18 years) exhibiting OSA-related signs, symptoms, and/or comorbidities, along with POSA on an in-lab attended polysomnography (PSG), were included. The included patients met the Mador definition of POSA (exclusive POSA): a supine AHI at least twice that in the non-supine position, with a non-supine AHI <5 [13]. Additionally, the PSG had to include at least 15 minutes of both supine and non-supine sleep. Exclusion criteria included previous exposure to PT, language barriers, and cognitive or psychiatric disorders.

Patients were screened through at-home recordings of their sleep positions using a Somnibel Pro® for two consecutive nights to confirm that the patient was sleeping in the supine position under their usual sleep conditions. Included patients, confirmed to have a supine sleeping position at home (supine sleep >25% of time in bed (TIB) on Somnibel Pro® during at least one of the two recording nights), were randomized using the REDCap randomization module to group A: Posiform® first, or Group B: Somnofit-belt® first. Patients were asked to use the two different PT devices consecutively (positional pillow, Posiform®, and vibrating belt, Somnofit-belt®), for two nights each, in their randomly assigned order, with concomitant recording of sleep position using the Somnibel Pro®. Patient questionnaires were completed after each of the four nights.

Devices

**Figure 1:**
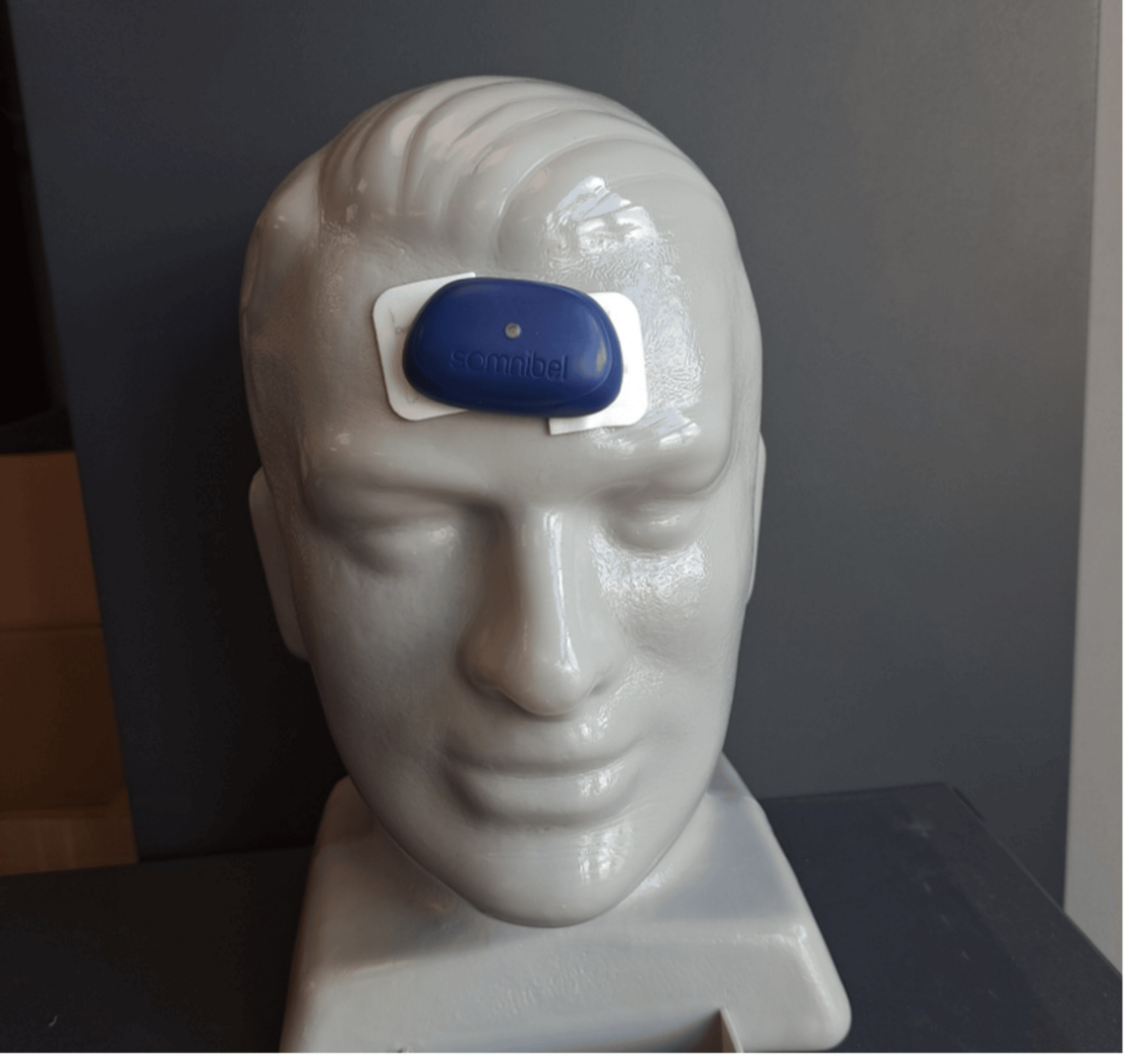
Somnibel Pro® device

Somnibel Pro® (Sibel SL company) is a vibrating device that includes an accelerometer/actimeter and is placed on the patient's forehead using a medical adhesive (Figure 1).

The device detects the supine head position (a surrogate for the supine body position) and starts vibrating until the patient changes from the supine to a non-supine position. In this study, the vibrating function was not enabled, but the device was used to obtain data related to body position during sleep [20]. When the recorded data are downloaded, a report can be generated showing the amount of time spent in the supine position during the night (percentage of time in bed, TIB). To accurately record time in the supine position while sleeping, the patient was instructed to place the device on their forehead at bedtime and remove it immediately after waking in the morning. For the analyses, the results for the night with the highest percentage of supine sleep across the three different conditions, two nights baseline, two nights with Posiform®, and two nights with Somnofit-belt®, were used.

**Figure 2:**
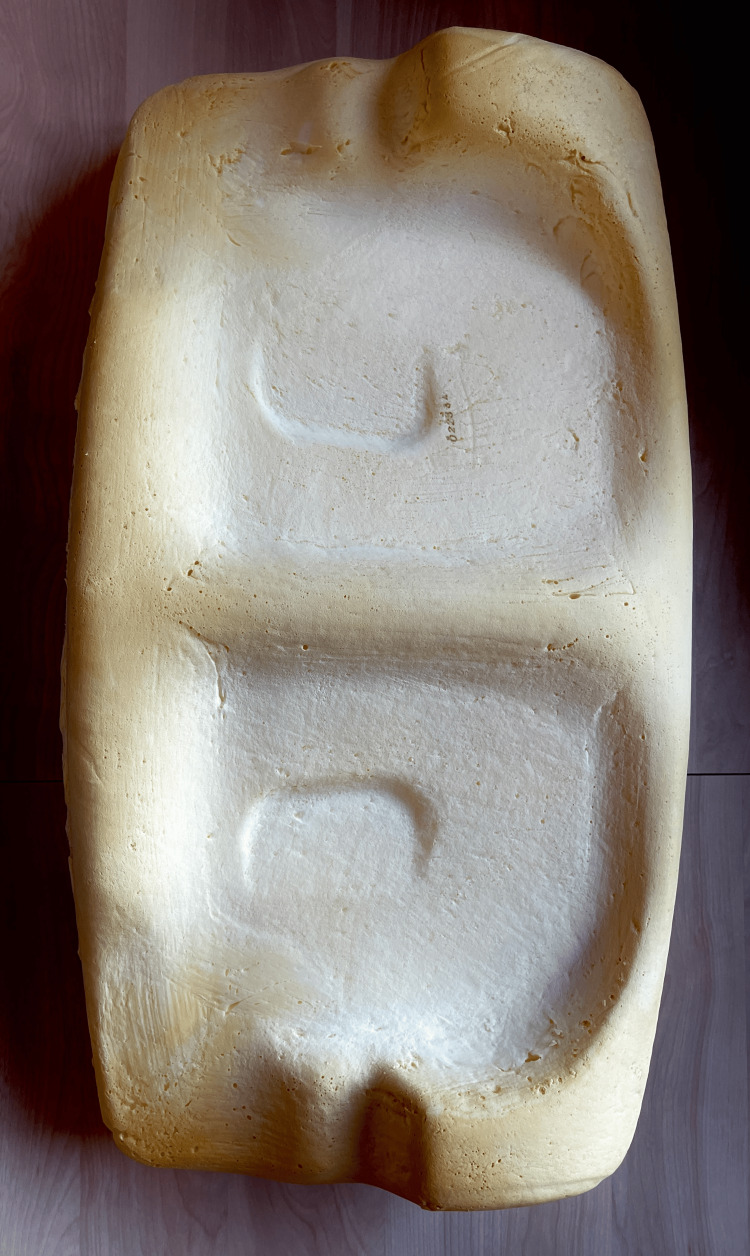
Posiform® pillow

Posiform® is a sleep positioning pillow (Oscimed S.A.) made of natural memory foam. It has a central ridge surrounded by two inclined, concave, and flattened surfaces, which force the patient to sleep on one of its sides to be comfortably positioned (Figure 2).

**Figure 3:**
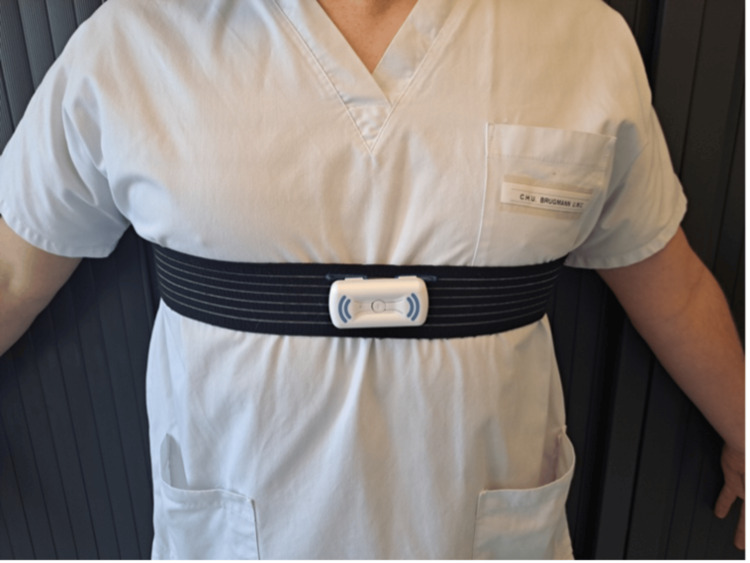
Somnofit-belt® device

The Somnofit-belt® (Oscimed S.A.) is a device attached to a belt, worn around the chest, that produces vibrations when the patient lies in a supine position (Figure 3). These vibrations continue at brief intervals until the patient turns onto their side.

Questionnaire

A seven-item questionnaire was used to assess each patient's opinion after each night spent with one of the devices (Table 1). Responses were evaluated on a visual analog scale (VAS), with zero corresponding to the worst score and 10 to the best score.

**Table 1:** Seven-item questionnaire with VAS scale VAS, visual analog scale

Questions	1	2	3	4	5	6	7	8	9	10
How was your sleep quality?										
Is the device easy to use?										
Is the device comfortable?										
Is the device effective for snoring?										
Is the device effective for sleep apnea?										
Would you pay for this type of treatment?										
What is your willingness to use it on a long-term basis?										

Answers are reported as the mean results of the two nights. Two other questions were also asked after the nights spent with the devices: "How was your sleep quality with the device (during the first night of use), compared to your usual sleep quality?" (with three possible answers: worse, equal, or better), and "How many times did you wake up during the night?" (answers are reported as the mean result of the two nights). The datasets generated and analyzed during the current study are available from the corresponding author upon reasonable request.

Statistical analysis

The statistical hypothesis was to demonstrate a 50% reduction in supine sleep positioning with both devices [19, 21]. A sample size of 52 subjects (26 in each group) was calculated as sufficient to achieve a power of 90% and accommodate a dropout rate of 10%. However, due to a high rate of screening failures, an interim analysis was conducted after the inclusion of 19 patients. As the difference between the treatments exceeded 50% and the pillow was ineffective, the decision was made to stop the study prematurely.

The entire group was compared to assess the efficacy of the belt and the pillow. The group that started with the Posiform® (Group A) and the group that started with the Somnofit-belt® (Group B) were compared. Within each group, values were compared between the devices. Descriptive statistics included calculating the median and quartiles (Q1 and Q3). After assessing the normality of the data, it was determined that parametric tests could not be applied. Therefore, a Mann-Whitney U test was used to compare the groups, and a Wilcoxon test was used to compare the effects of the devices within each subgroup. For categorical data, a chi-square test was applied. A p-value threshold of less than 0.05 was considered significant. Statistics were compiled using Python 3.12 with the SciPy version 1.11.4 statistical module.

## Results

**Figure 4:**
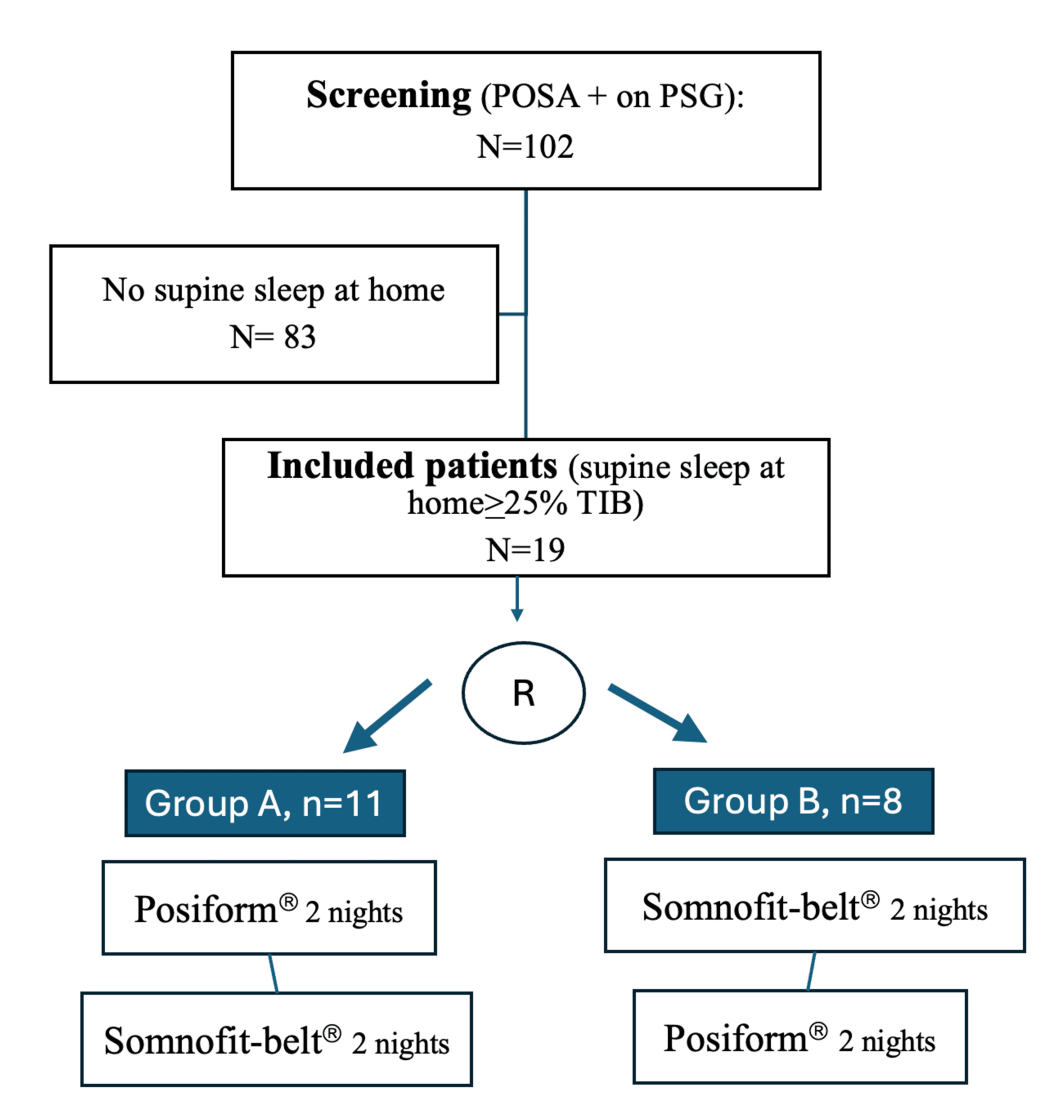
Flow chart of the study POSA, positional obstructive sleep apnea; PSG, polysomnography; TIB, time in bed; R, randomization

Between September 2020 and December 2023, a total of 102 patients exhibiting POSA on PSG performed in the sleep lab were screened for inclusion. The study duration was 26 months. In all patients, the non-supine AHI was <5 per hour. Of these, 19 patients (19%) were included, and 83 patients (81%) were excluded because they did not demonstrate supine sleep of at least 25% at home. A flow chart is illustrated in Figure 4.

The median supine TIB (min-max) was 9.15% (0.4-24.7%) for non-eligible patients and 32.1% (25.0-52.4%) for the 19 included patients. Included patients were randomized to sleep with Posiform® first (n=11, 58%) (group A) or Somnofit-belt® first (n=8, 42%) (group B). Patient characteristics are summarized in Table 2.

**Table 2:** Demographics and sleep characteristics of included patients Group A: Posiform® first, group B: Somnofit-belt® first ^(1)^Mann-Whitney U test ^(2)^Chi-square (X2) test BMI, body mass index; AHI, apnea-hypopnea index; TST, total sleep time; TIB, time in bed; IQ, interquartile; PSG, polysomnography

Variable (min-max)	Group A (n=11), median (IQ1;IQ3) or frequency (%)	Group B (n=8), median (IQ1;IQ3) or frequency (%)	p-value (p<0.05=significant value)
Age, years	52.5 (51.25;56.25)	51.0 (39.0;60.5)	0.7725^(1)^
Sex, male, n (%)	8 (72.7%)	7 (87.5%)	0.4835^(2)^
BMI, kg/m^2^	27.75 (26.3;31.48)	25.9 (23.7;28.35)	0.2153^(1)^
PSG			
AHI	16.7 (12.18;19.17)	17.4 (12.8;19.05)	0.9678^(1)^
AHI supine	37.25 (30.55;40.33)	32.4 (26.05;52.7)	0.8404^(1)^
TST (minutes)	328.5 (293.38;377.75)	363.5 (305.0;384.75)	0.6201^(1)^
TST supine (minutes)	92.1 (67.6;231.7)	136.7 (88.0;196.5)	0.6574^(1)^
TST supine (%)	29.16 (23.17;61.3)	37.12 (32.28;50.38)	0.4920^(1)^
TST non-supine (minutes)	221.5 (184.95;269.5)	252.0 (135.0;267.0)	0.7725^(1)^
Somnibel Baseline TIB supine (%)	36.9 (30.22;44.5)	32.1 (30.6;39.05)	0.6796^(1)^

**Figure 5:**
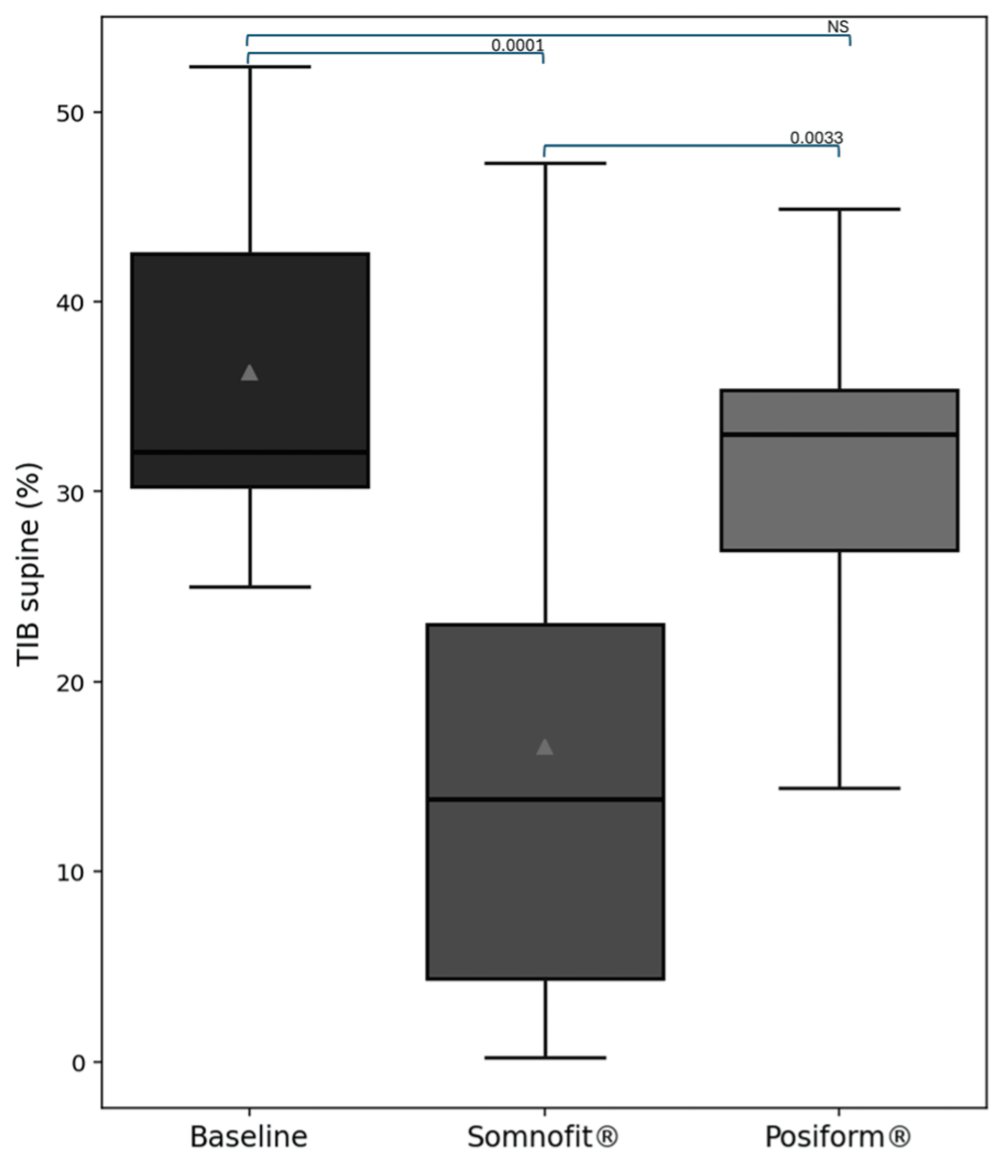
Changes in supine TIB with the use of Somnofit-belt® or Posiform® TIB, time in bed

For the group as a whole, the Somnofit-belt® was significantly more effective than the Posiform®, providing a reduction in supine TIB of 26.4% vs. 7% for the Posiform®, p=0.0033 (Table 3 and Figure 5). No significant differences were observed between the two devices in the satisfaction questionnaire (Table 4).

**Table 3:** Impact of Posiform® and Somnofit-belt® on sleep characteristics for the whole group TIB, time in bed

Somnibel	Baseline, n=19, median (IQ1;IQ3)	Posiform®, median (IQ1;IQ3)	Somnofit-belt®​​​​​​​, median (IQ1;IQ3)	p-value for Wilcoxon test (p<0.05=significant value)
% TIB supine	32.1 (30.25 ;42.55)	33.0 (26.95;35.35)	13.8 (4.35;23.0)	0.1000 ( Posiform®​​​​​​​), 0.0001 (Somnofit-belt®​​​​​​​)
Delta TIB		-7.0 (-4.35;11.1)	-26.4 (-8.2;-30.2)	0.0033

**Table 4:** Satisfaction questionnaire results for Posiform® and Somnofit-belt® for the whole group (N=19)

Questionnaire	Posiform®, median (IQ1;IQ3)	Somnofit-belt®​​​​​​​, median (IQ1;IQ3)	p-value for Wilcoxon test (p<0.05=significant value)
How was your sleep quality?	4.0 (2.0;6.0)	6.0 (2.0;6.0)	0.1754
Is the device easy to use?	10.0 (5.0;10.0)	10.0 (8.0;10.0)	0.1579
Is the device comfortable?	6.0 (3.0;6.0)	6.0 (4.0;8.0)	0.4386
Is the device effective for snoring?	5.0 (1.5;6.5)	7.0 (4.0;8.0)	0.1060
Is the device effective for sleep apnea?	4.0 (1.0;6.0)	6.0 (4.0;8.0)	0.1363
How many times did you wake up during the night? (mean of two nights)	2.5 (1.0;3.5)	2.5 (1.5;3.75)	0.9497
Would you pay for this treatment?	2.0 (0.0;6.0)	6.0 (2.0;8.0)	0.1069
What is your willingness to use it on a long-term basis?	2.0 (0.0;7.0)	4.0 (2.0;6.0)	0.2447

The differences in subjective sleep quality and nighttime symptoms (Posiform® or Somnofit-belt® vs. baseline) were not significant (p>0.05).

## Discussion

In this small pilot study comparing PTs for POSA, there is a trend toward greater efficacy of the vibration belt compared to the positional pillow in reducing supine sleep.

To our knowledge, this is the first study comparing two different PTs for POSA. Previous studies have compared PT to mandibular advancement devices (MAD) or CPAP, but not head-to-head PT devices.

This prospective randomized crossover study highlights the fact that POSA is infrequent in the home setting, observed in only 19% of patients diagnosed with POSA in the sleep lab. Buyse et al. previously reported that supine sleep is less prevalent at home compared to the sleep lab in a cohort of 51 patients treated with SPT [14]. The prevalence of POSA is very high in sleep labs, estimated to be 39%-50% of OSA diagnoses based on large cohorts [22-24]. However, data from home sleep recordings have suggested a lower prevalence. For example, in the HypnoLaus sleep cohort, exclusive POSA diagnosed at home represented 36% of all 1220 OSA patients (AHI>5) [25]. Another study observed a lower prevalence of 23% in 434 patients with PSG at home [26].

The differences in prevalence between home and sleep lab conditions may be explained by several factors. In the sleep lab, recording conditions are adversely affected by the increased weight of the PSG recording device (generally worn on the chest) and the unusual sleep environment, which can provoke more supine sleep and respiratory events related to increased arousability [27]. In our study, the low prevalence of supine sleep at home may be related to the measurement method we used to estimate supine sleep time. We used a device placed on the forehead, which may have underestimated or overestimated the actual supine sleep time at home, as the position of the head may not have followed that of the trunk during the night. The type and quality of the pillow may also have influenced the head position. This method's limitations contributed to the low prevalence of POSA under usual sleep conditions, which led to recruitment difficulties and premature study discontinuation. Nevertheless, we were able to confirm significant differences in effectiveness between the tested PT devices. As confirmed in previous studies, this study found that the modern vibrating belt, the Somnofit-belt®, was effective in significantly reducing supine sleep, suggesting that the device was able to normalize AHI in this exclusively POSA population. Recent vibrating PT devices have proven effective, well-tolerated, and led to good long-term adherence [18, 28].

However, beyond efficacy, the comfort of use scored only 6/10 on the VAS, and the willingness to use it long-term was low (4/10). Based on clinical experience, we know that some patients are resistant to vibrations, while others prefer to sleep supine and become anxious after experiencing numerous vibrations during the night, ultimately discontinuing vibrating PT use [14, 15]. In such cases, PT is not the treatment of choice for POSA, and patients should consider switching to more conventional therapies, such as MAD or CPAP [16].

In this study, the positional pillow (Posiform®) was not able to significantly reduce supine sleep in POSA patients. The methods work on different principles. The therapeutic mechanisms of the positional pillow differ greatly from vibrating belts. The positional pillow has a central ridge that prevents the individual from sleeping in the supine position and stimulates lateral positioning of the head during sleep. On the other hand, the vibrating belt activates when the individual sleeps in the supine position. The problem with the pillow is that it is not “attached” to the patient, allowing them to easily move and sleep beside the pillow, losing its effect. Other types of pillows, such as the SONA pillow, which has a space for the arm under the head while sleeping on the side [29], have been tested in very small series and may be less likely to be avoided. This pillow appears effective for mild to moderate POSA, but data are limited and have not been reproduced in larger cohorts.

In another series of 25 patients with snoring and POSA, Chen et al. [30] tested a head-positioning pillow (Power Sleep®) over two nights. Like the Posiform®, the Power Sleep® encourages the head to turn into the lateral sleep position, as its median portion is narrower than the lateral sleep part. The study showed a 33.3% reduction in snoring severity. However, sleep position and AHI were not assessed. In a study by Newell et al. [19], 28 POSA patients used the Posiform®. Patients were assessed at baseline, and again at one and six months with PSG. Statistically significant changes were observed in TST supine, from 52% to 20%, and in AHI, from 12.1 to 6. However, supine sleep was not completely abolished. Neck pain was reported in one patient, leading to treatment interruption. In this study, the satisfaction rate and patients’ willingness to pay for the pillow and use it long-term were much lower than in the Newell study, possibly because patients had access to comparative therapies and due to the small number of patients included.

For many patients, the main reported outcome measure is snoring [19, 28, 30]. While both investigated PT devices displayed subjective alleviation of snoring, the improvement with the Somnofit®-Belt was more pronounced, though not significantly different.

Given the differences between devices, further studies are needed to compare PT devices head-to-head (e.g., different types of vibrating belts and pillows). Improvement in symptoms and/or quality of life in POSA when treated long-term with vibrating belts or PT should also be studied.

Limitations

The most important limitation of this study is the small sample size. Recruitment difficulties were twofold. On the one hand, due to the COVID-19 pandemic, the number of PSGs performed decreased, and fewer cases of positional OSA were detected. On the other hand, once enrolled, it became apparent that the real prevalence of POSA, when assessed in the home setting, was extremely low. The second limitation is that PT efficacy was not measured by follow-up PSG, which resulted in the absence of AHI measurement. TST (supine/non-supine) was also estimated through TIB measurement using the Somnibel Pro®. TIB supine is only a surrogate for therapeutic effectiveness. Additionally, we did not assess the evolution of POSA-related symptoms due to the short duration of the therapeutic trial (2x2 days), which obviously had no major impact on them. A final limitation was the lack of follow-up data on the long-term use of both PT devices. However, the main strength of the study was its design as a randomized crossover study.

## Conclusions

This small prospective randomized crossover pilot trial comparing two PTs for POSA suggests that the vibrating belt is more effective in reducing supine sleep than the positional pillow. Furthermore, POSA appears to be widely overdiagnosed in a hospital PSG setting compared to an assessment in a home setting. Given the small sample size, further studies are needed to confirm these results and assess patient-reported outcomes during the long-term use of PT.
